# A Case Report of Falciform Ligament Appendagitis

**DOI:** 10.7759/cureus.51965

**Published:** 2024-01-09

**Authors:** Marta Manso, Lisa Agostinho

**Affiliations:** 1 Department of Radiology, Hospital Beatriz Ângelo, Lisbon, PRT

**Keywords:** acute abdomen, focal fat infarction, abdominal pain, appendage torsion, falciform ligament appendagitis

## Abstract

Falciform ligament appendagitis is an extremely rare form of intra-abdominal focal fat infarction. It usually presents with vigorous abdominal pain and mimics other more common acute abdominal pain-associated diseases. Better recognition of this entity avoids misdiagnoses and unnecessary surgical treatment. We present the case of a 73-year-old woman admitted to the emergency department for abdominal pain, nausea, and vomiting. She had a fever and a diffuse tender abdomen with upper right quadrant pain. Laboratory investigation showed leukocytosis and high C-reactive protein. CT revealed a heterogeneous increased density of fat adjacent to the falciform ligament. Falciform ligament appendagitis was diagnosed and antibiotic and anti-inflammatory treatment resulted in complete recovery. This case highlights the need to raise awareness and better recognize falciform ligament appendagitis to avoid unnecessary surgical interventions.

## Introduction

Intra-abdominal focal fat infarction occurs more commonly in the epiploic appendages or greater omentum and rarely involves the perigastric ligaments (gastrohepatic, gastrosplenic, and falciform). The falciform ligament is a peritoneal fold dividing the left and right hepatic lobes and torsion is extremely rare in this location [1,2]. The clinical presentation is non-specific with severe abdominal pain and raised inflammatory markers. Computed tomography (CT) has a major diagnostic role allowing for an accurate differential diagnosis from other more common acute abdominal diseases, avoiding misdiagnosis and unnecessary surgical interventions [2-4].

We report the case of a 73-year-old woman presenting with abdominal right upper quadrant pain, nausea, and vomiting. Fever and abdominal tenderness were noted. Laboratory tests showed elevated inflammatory parameters and mild cytocholestasis. CT revealed falciform ligament appendagitis and antibiotic and anti-inflammatory analgesia were started with clinical and laboratory improvement.

## Case presentation

A 73-year-old female was admitted to the emergency department with three days of existing fever, vigorous continuous right upper quadrant pain, nausea, and vomiting. She had no diarrhea or exanthema. She had a previous history of hypothyroidism, type 2 diabetes, dyslipidemia and penicillin, and cephalosporin allergy.

At physical examination, the patient showed abdominal diffuse mild tenderness with upper right quadrant pain without signs of peritoneal stimulation. She had a normal body mass index.

Laboratory tests showed leukocytosis (16260 WBCs/mm^3^, 84% neutrophils), elevated C-reactive protein (CRP) (52 mg/dL), mild cytocholestasis (alanine aminotransferase 70 units/L, gamma-glutamyl transferase 185 units/L, alkaline phosphatase of 174 units/L) with normal bilirubin (BR 0.86 mg/dL) and normal lactate level (1.2 mmol/L).

**Figure 1:**
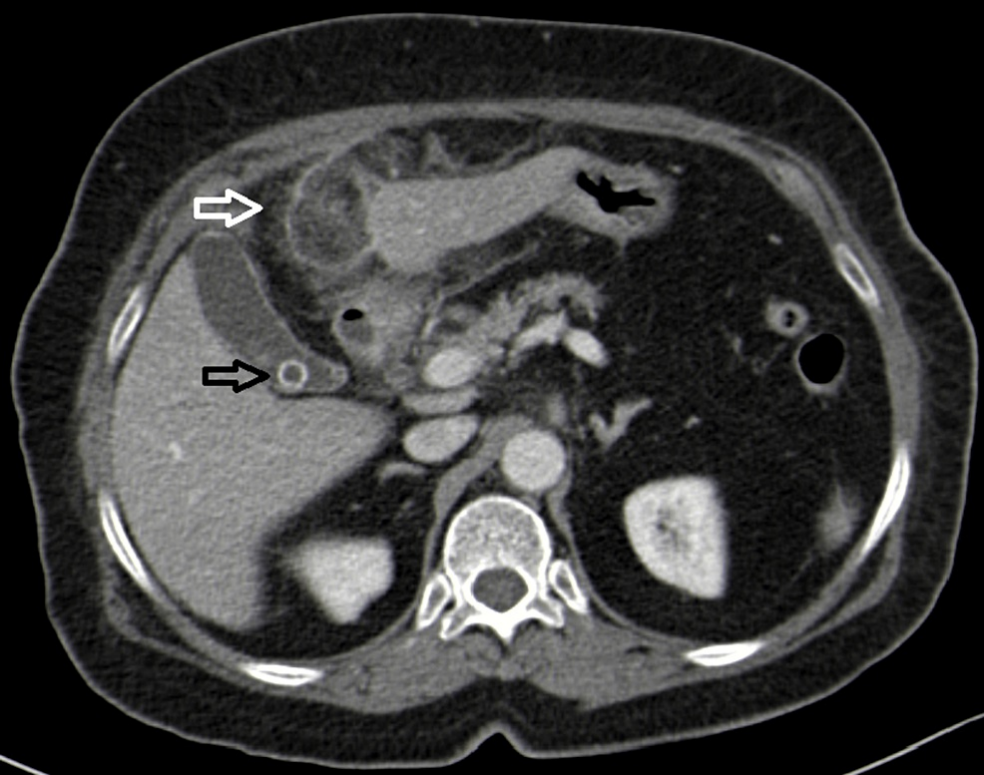
Computed tomography axial plane showing heterogeneous increase density of fat and hyperattenuating rim sign adjacent to falciform ligament, compatible with falciform ligament appendagitis (white arrow). Vesicular lithiasis with normal gallbladder appearance can also be seen (black arrow).

**Figure 2:**
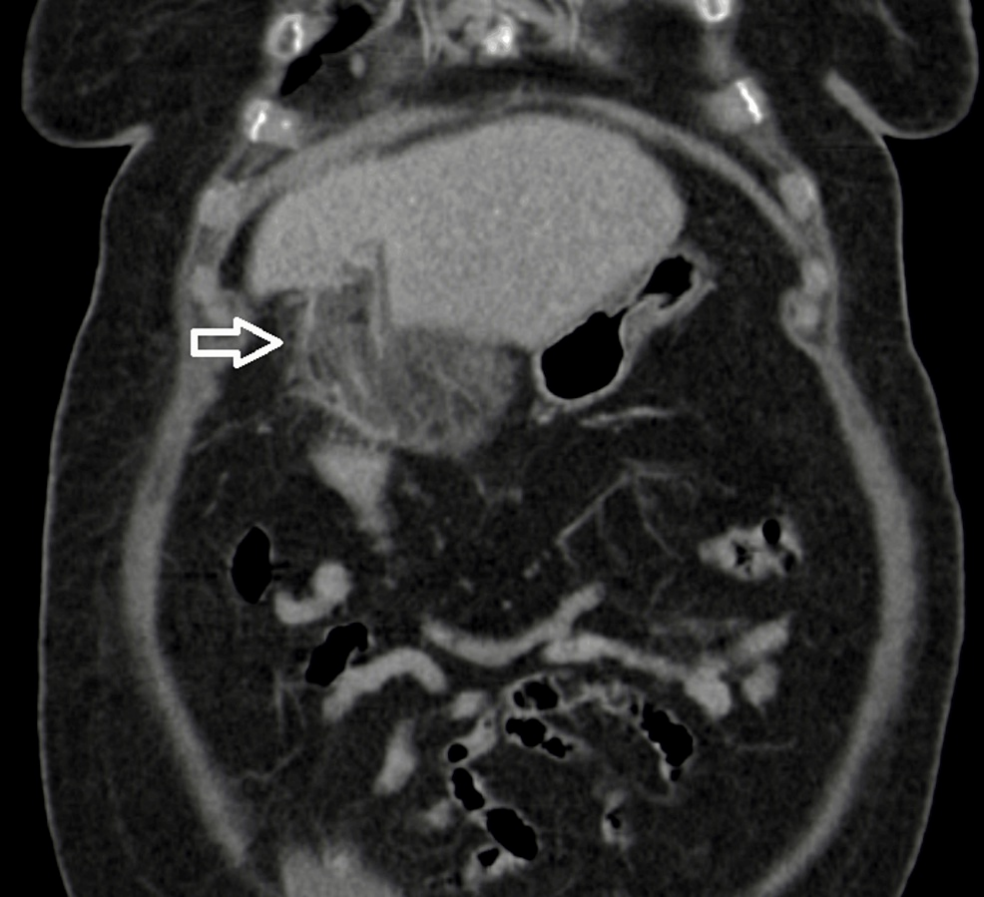
Coronal computed tomography reconstruction demonstrating the infarcted appendage (white arrow).

**Figure 3:**
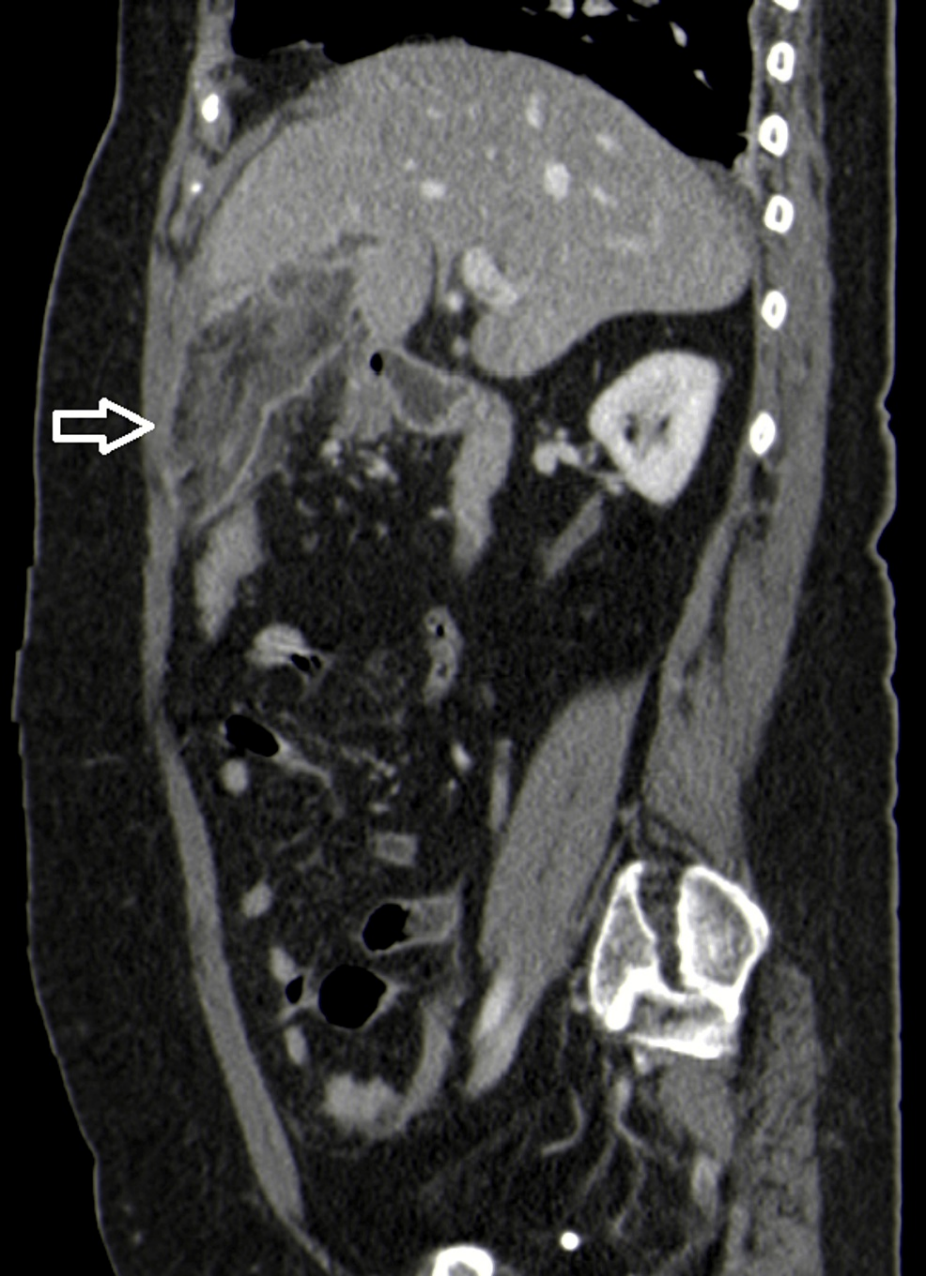
Sagittal computed tomography reconstruction showing the infarcted appendage (white arrow).

Contrast-enhanced CT was performed and revealed a heterogeneous increase density of fat adjacent to the falciform ligament suggesting fat necrosis, compatible with falciform ligament appendagitis (Figures 1-3).

A hyperattenuating rim sign could also be seen surrounding this area. Vesicular lithiasis was noted. There was no bile duct dilatation, pneumoperitoneum, enteric distension, or free fluid in the peritoneal cavity.

The patient was started on an antibiotic (ciprofloxacin) and anti-inflammatory analgesia and clinical and laboratory improvement (CRP 2 mg/dL) was shown after seven treatment days.

## Discussion

Torsion and infarction of the falciform ligament is an exceedingly rare form of intra-abdominal focal fat infarction involving torsion of fatty appendage of the falciform ligament, with less than 20 cases documented with imaging studies [2].

The falciform ligament is a double fold of peritoneum separated by extraperitoneal fat that divides the left and right liver lobes [1]. The falciform ligament contains the ligamentum teres which is a cord-like remnant of the obliterated umbilical veins, paraumbilical veins, and variable amounts of extraperitoneal fat [3,5]. It receives arterial supply from a vessel coming from the left inferior phrenic artery and the middle segmental artery of the liver [3].

Falciform ligament appendagitis is caused by torsion of the long lipomatous appendage leading to ischemia and ultimately aseptic fat necrosis [3]. It is slightly more frequent in males than in females (ratio close to 1:1) with a median age of 59.5 years and the only documented risk factor is obesity, specifically increased abdominal visceral adipose tissue although limited literature is available [3,5].

Clinical presentation includes right upper quadrant and epigastric pain, usually non-radiating, low-grade fever, and leukocytosis [3,5].

Isolated inflammation occurs but inflammation can also be associated with infection of adjacent structures such as gallbladder, liver, peritoneum, and thoracic or abdominal wall and these should be sought for [5].

Other rare pathologies of falciform ligament include internal hernia through congenital defects, cysts, and lipomas, all presenting with similar clinical symptoms [2].

Due to its rarity, falciform ligament appendagitis is very difficult to diagnose preoperatively and is easily confused with other more common causes of acute abdominal pain including cholecystitis, appendicitis, hepatitis, or diverticulitis [5].

On ultrasound, falciform ligament appendagitis appears as an oval hyperechoic, noncompressible, heterogenous mass but in mild cases can be unnoticed. CT has a major diagnostic and differential diagnostic role, appearing as an oval area of heterogeneous fat attenuation surrounded by a thin peripheral rim of hyperattenuation that represents an adjacent inflamed peritoneum. A central area of hyperattenuation can sometimes be seen and is known as the dot sign representing venous thrombosis. Associated inflammatory changes in the adjacent fat planes can also occur [2-4].

Falciform ligament appendagitis is a self-limiting disease that requires no surgical treatment and usually resolves with conservative treatment in 3-14 days [1,2]. Rare complications include adhesions, peritonitis, abscess formation, and calcified peritoneal loose bodies [2].

## Conclusions

Falciform ligament appendagitis is an extremely rare location of intra-abdominal focal fat infarction and poses a diagnostic challenge due to its rarity and unspecific presentation, common to other more frequent abdominal acute diseases.

CT is the imaging method of choice to identify this condition and has a major role in differential diagnosis. Better awareness of this type of torsion allows the inclusion of this rare entity in the differential diagnosis of acute abdomen avoiding missed or delayed diagnosis.
